# Posttraumatic Psychiatric Disorders and Resilience in Healthcare Providers following a Disastrous Earthquake: An Interventional Study in Taiwan

**DOI:** 10.1155/2017/2981624

**Published:** 2017-10-09

**Authors:** Ya-Ting Ke, Hsiu-Chin Chen, Chien-Ho Lin, Wen-Fu Kuo, An-Chi Peng, Chien-Chin Hsu, Chien-Cheng Huang, Hung-Jung Lin

**Affiliations:** ^1^Department of Nursing, Chi-Mei Medical Center, Tainan, Taiwan; ^2^Graduate Institute of Nursing, Kaohsiung Medical University, Kaohsiung City, Taiwan; ^3^Bachelor Program of Senior Service, Southern Taiwan University of Science and Technology, Tainan, Taiwan; ^4^Department of Psychiatry, Chi-Mei Medical Center, Tainan, Taiwan; ^5^Department of Emergency Medicine, Chi-Mei Medical Center, Tainan, Taiwan; ^6^Department of Biotechnology, Southern Taiwan University of Science and Technology, Tainan, Taiwan; ^7^Department of Environmental and Occupational Health, College of Medicine, National Cheng Kung University, Tainan, Taiwan; ^8^Department of Occupational Medicine, Chi-Mei Medical Center, Tainan, Taiwan; ^9^Department of Geriatrics and Gerontology, Chi-Mei Medical Center, Tainan, Taiwan; ^10^Department of Emergency Medicine, Taipei Medical University, Taipei, Taiwan

## Abstract

**Background:**

Posttraumatic psychiatric disorders (PTPDs) are common in disaster workers; however, their incidence and resilience in healthcare providers (HCPs) following a disastrous earthquake are still unclear. Therefore, we conducted an interventional study to clarify this issue.

**Methods:**

After a medical response to the scene of a collapsed huge building, we conducted an assessment of the HCPs using an immediate self-administered questionnaire and a follow-up questionnaire 1 month later. Psychological support after the operation was implemented. We performed analysis of the risk for PTPDs and comparison between immediate and follow-up questionnaires.

**Results:**

The mean age (standard deviation) of the HCPs was 32.7 (5.2) years, with 33.5 (5.8) years for nurses and 32.4 (4.4) years for physicians. The proportion of females among the nurses and physicians was 94.3% and 12.5%, respectively. In total, 16.4% (11/67) of HCPs fit the criteria of PTPDs. Nurses had a trend of higher incidence than physicians. Female HCPs had a trend of higher incidence than male HCPs. After intervention, none of the HCPs reported PTPDs in the follow-up questionnaire (*p* < 0.05).

**Conclusion:**

This study delineated that PTPDs were common in HCPs following medical response to an earthquake; however, the resilience was good after the early intervention.

## 1. Introduction

Early posttraumatic psychiatric disorders, a not-well defined group of diseases, including acute stress disorder (ASD), probable depression, and increased tobacco use, are common in disaster workers, including healthcare providers (HCPs) [1–3]. Posttraumatic psychiatric disorders are different from posttraumatic stress disorder (PTSD), which is a specific term that symptoms must last more than a month and be severe enough to interfere with relationships or work [3]. The study on the 9/11 World Trade Center disaster workers reported that nearly 15% of disaster workers had probable ASD, 26% had probable depression, and more than half of tobacco users increased their tobacco use [1]. A 10-year longitudinal study reported that there was a persistent mental health disturbances including PTSD, anxiety, depression symptoms, and sleeping problems in the disaster victims [3]. The disaster-related ASD and depression may cause functional impairment in the HCPs, which in turn affects the quality of patient care [1]. Therefore, prevention, early recognition, and intervention, including the following (1) before work: consideration of comfort level with this type of work and current health, family, and work circumstances in the HCPs; (2) during work: recognition of common and extreme stress reactions and ability of organizations to reduce the risk of extreme stress to HCPs, taking care of themselves by HCPs, and supports and policies by the organizations (i.e., limiting shifts, rotation of providers, mandatory time-off, identifying enough providers at all levels, encouraging peer partners and peer consultation, monitoring providers who meet certain high-risk criteria, establishing supervision, case conferencing, and staff appreciation events, and conducting training on stress management practices); and (3) after work: a readjustment period upon returning home and making personal reintegration a priority for a while in the HCPs, are suggested for the high-risk group such as all the disaster workers [1, 4].

Resilience is defined as the capacity to respond to stress in a healthy way such that the goals are achieved at minimal psychological and physical cost, which is key to enhancing the quality of care and sustainability of the healthcare workforce [5]. Several factors may affect resilience, including individual, community, and institutional factors [5]. For example, individual factors include self-awareness, self-monitoring, and self-regulation [5]. Community factor includes the general public's sympathy for the HCPs [5]. Institutional factor includes training and continuing education in the hospitals or medical schools [5]. Resilience has been recognized as an important component of training in disaster workers [6]; however, resilience in HCPs as well as posttraumatic psychiatric disorders after earthquake rescue has not been well studied. On February 6, 2016, an earthquake measuring 7.0 occurred in Southern Taiwan, which resulted in 117 deaths and 513 people wounded [7]. Sixty-seven HCPs from Chi-Mei Medical Center (CMMC) were sent for the medical response. We conducted a prospective study to analyze the incidence of posttraumatic psychiatric disorders and resilience in HCPs following the intervention after the disastrous earthquake. Comparisons for age subgroups, sex, educational levels, marital status, years of service, and types of occupation between nurses and physicians were also performed.

## 2. Methods

### 2.1. Study Setting, Design, and Participants

The earthquake caused the collapse of a huge 16-floor building in Tainan city and resulted in 115 deaths and 96 people wounded, making it the most serious disaster among single-building collapses in the history of Taiwan. CMMC is the largest tertiary medical center with a total of 1,276 inpatient beds and an 80-bed ED staffed with board-certified emergency physicians who provide emergency care to approximately 145,000 patients per year [8]. Being located near the collapsed building, CMMC sent 67 HCPs, including 35 nurses and 32 physicians, for the on-site rescue. All the 67 HCPs worked in an 8 h shift. In addition to the HCPs from CMMC, seven local disaster medical assistance teams were deployed for the field operation [7]. Meetings were held on the scene twice a day to proactively formulate team-oriented protocols for the on-site HCPs, emergency medical technicians, and administrative associates [7]. For the occupational safety, all the mandatory airway protection equipment, including at least N95 respirator masks, was provided to all the workers to prevent airway complications from the deconstruction of the collapsed building [7]. The mean age (standard deviation) of the total HCPs was 32.7 (5.23) years, while that of the nurses and physicians was 33.5 (5.8) years and 32.4 (4.4) years, respectively (Table 1). The range of age in total HCPs, nurses, and physicians was 23–45 years, 23–43 years, and 27–45 years, respectively. About 57% of the HCPs were aged 30–39 years. Nurses had a higher female percentage (94.3%) than physicians (12.5%). The range of years of services in total HCPs, nurses, and physicians was 1–21.4 years, 1–21.4 years, and 2–20 years, respectively. Years of service were 9.0 (6.0) years in the total HCPs and were longer in the nurses than in the physicians (11.2 [5.9] years versus 6.5 [5.2] years). More than 86% of the HCPs had a bachelor degree. About half of the HCPs were unmarried.

### 2.2. Intervention for Preventing HCPs from Posttraumatic Psychiatric Disorders

Since the workers handling the seriously injured or dead victims might suffer from psychological distress [1], psychological support, including psychotherapists and psychiatrists providing on-site debriefing courses and minilectures for the HCPs to improve awareness of mental health, was also provided [7]. In addition, physical therapists provided muscle and mental relaxation programs on the scene [7]. After the operation, all the workers were encouraged to express their feelings about the disaster to relieve their stress during and after the debriefing. A 1-year follow-up program was developed to monitor posttraumatic psychiatric disorders by psychiatrists [7]. The intervention was implemented according to the guideline of Psychological First Aid for Provider Care [4].

### 2.3. Instruments for Evaluation of Posttraumatic Psychiatric Disorders: Immediate Self-Administered Questionnaire for the HCPs after the Medical Response and Follow-Up Questionnaire 1 Month Later

**Figure 1:**
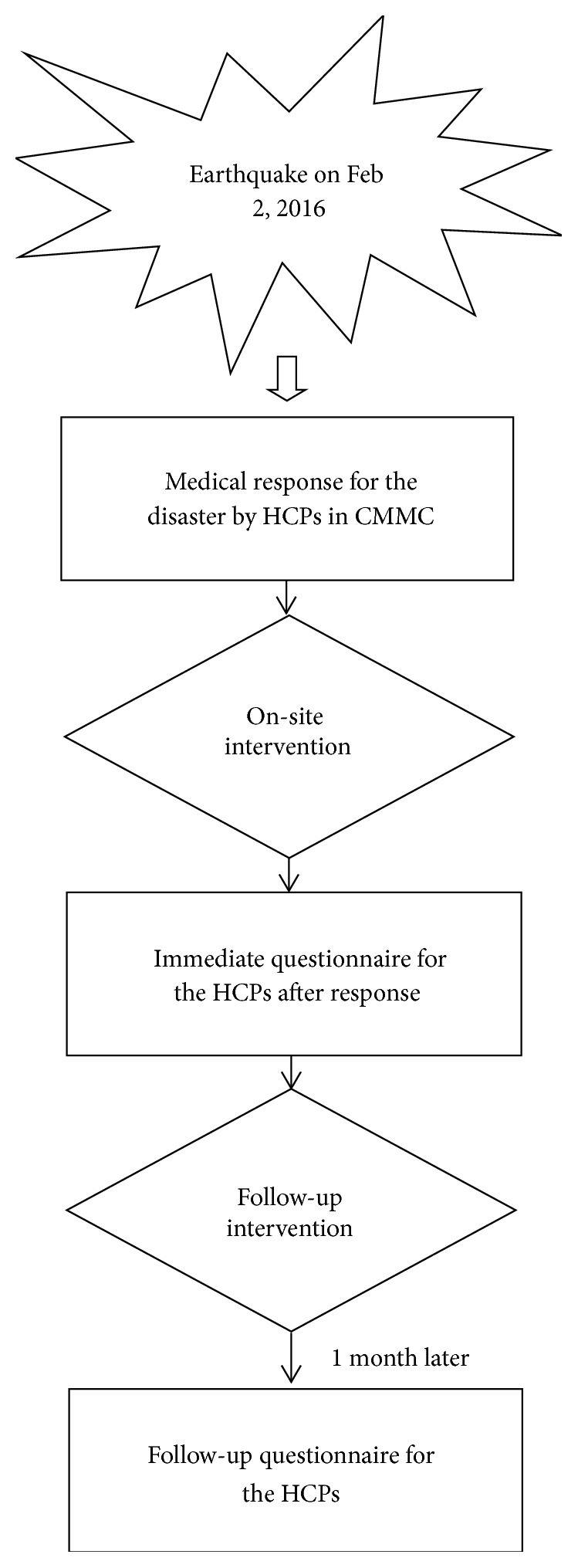
Flowchart of this study. HCPs, healthcare providers; CMMC, Chi-Mei Medical Center.

A head nurse in CMMC initiated a questionnaire study in the CMMC HCPs immediately after the medical response (Figure 1). There is no validated instrument specific for evaluation of posttraumatic psychiatric disorders in HCPs after a disaster response in the literature. Therefore, the questionnaire was constructed by a psychiatrist containing the following symptoms that were screened for suspected posttraumatic psychiatric disorders based on the criteria of ASD and the situation at that time: (1) recurrent and intrusive distressing recollections of the event, including images, thoughts, or perceptions; (2) tachycardia; (3) muscle tension; (4) difficulty relaxing; (5) difficulty falling or staying asleep; (6) feeling fear; (7) feeling guilty; (8) needing help after the medical response; and (9) needing to talk with someone in private [9]. Items (1)–(7) are some of the criteria of ASD, which is based on the Diagnostic and Statistical Manual of Mental Disorders, Fourth Edition, Text Revision (DSM-IV-TR) [9]. The eighth and ninth items are the active needs from the HCPs. There is a yes or no option for each item. Administrative chiefs responsible for the HCPs actively made conversation with the HCPs who reported any positive symptoms in the questionnaire or any abnormal report from the HCPs themselves or their coworkers and referred them to psychiatrists for further treatment, if necessary. After 1 month, a follow-up questionnaire study was conducted in the same 67 HCPs.

### 2.4. Data Collection, Definition of Posttraumatic Psychiatric Disorders, and Resilience

Four researchers collected the data prospectively. Suspected case for posttraumatic psychiatric disorders was defined as any reported symptom in the questionnaire. There is no consensus about the definition of resilience in the literature. Connor and Davidson ever proposed a Connor-Davidson Resilience scale (CD-RISC) and Friborg et al. proposed Resilience Scale for Adults (RSA) for measuring resilience [10, 11]; however, both CD-RISC and RSA are not suitable for this study because they are not designed for the acute situation as this study. Therefore, for the unique feature in this study, resilience was defined as a recovery from any reported symptom (i.e., positive in the immediate questionnaire and negative in the follow-up questionnaire).

### 2.5. Ethics Statement

This study was conducted strictly according to the Declaration of Helsinki and the requirements of the institutional review board at CMMC. Because the questionnaire study was a routine practice for HCPs, the informed consent of the participants was waived.

### 2.6. Statistical Analysis

Independent samples* t*-test was used for continuous variables (age and years of service) and Pearson chi-square test was used for categorical variables (age subgroup, sex, education, marital status, questionnaire, and occupation) in the comparison of posttraumatic psychiatric disorders by cases and controls (with posttraumatic psychiatric disorders versus without posttraumatic psychiatric disorders). McNemar's test was used for the comparison of posttraumatic psychiatric disorders between immediate questionnaire and follow-up questionnaire. SPSS software version 16.0 was used for the analysis. The significant level was set at 0.05 (two-tailed).

## 3. Results

All the 67 HCPs completed the self-administered immediate and follow-up questionnaires. The most common symptom reported by the HCPs was recurrent and intrusive distressing recollections of the event, including images, thoughts, or perceptions (13.4%), followed by tachycardia (4.5%), difficulty relaxing (4.5%), and difficulty falling or staying asleep (1.5%). There was no significant difference in all the items between the nurses and physicians. The incidence of posttraumatic psychiatric disorders was 16.4% (11/67) in all the HCPs. Nurses had a trend of higher incidence of posttraumatic psychiatric disorders than physicians (22.9% versus 9.4%).

Univariate analysis of posttraumatic psychiatric disorders revealed that there was a trend of higher percentage of female HCPs in the posttraumatic psychiatric disorders group (81.8%) (*p* = 0.095) (Table 2). There was no significant difference in age, years of service, occupation, education, and marital status between HCPs with and without posttraumatic psychiatric disorders.

**Figure 2:**
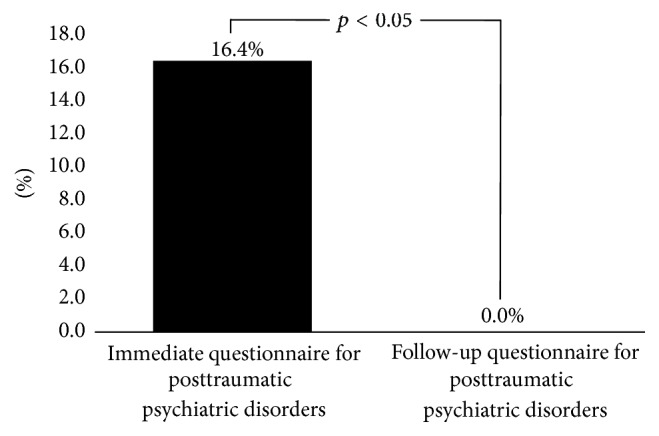
The comparison of posttraumatic psychiatric disorders between immediate and follow-up questionnaires. *y*-axis indicates the percentage of participants with any positive symptom in all participants.

The basic characteristics of the HCPs with posttraumatic psychiatric disorders are shown in Table 3. The years of service ranged from 1 to 20.4 years (Table 3). Four HCPs were unmarried (36.4%, 4/11). A 32-year-old male physician had four positive items. This physician was referred to a psychiatrist for counseling. After the intervention, the follow-up questionnaire 1 month later revealed no symptoms among the total HCPs (Figure 2).

## 4. Discussion

This study showed that 16.4% of HCPs had posttraumatic psychiatric disorders due to the medical response for the huge building collapse caused by the earthquake. The most common reported symptom was recurrent and intrusive distressing recollections of the event, including images, thoughts, or perceptions. Nurses had a trend of higher incidence of posttraumatic psychiatric disorders than the physicians. There was a higher percentage of female HCPs in the posttraumatic psychiatric disorders group (81.8%); however, the difference was not significant, even in the other variables, including age, years of service, occupation, education, and marital status. After the interventions, including on-site debriefing and psychological support programs and follow-up care and referral to the psychiatrist as necessary, none reported the symptoms listed in the questionnaire 1 month later.

Results of this study show that early posttraumatic psychiatric disorders are common in disaster workers. An earlier study about airplane crash reported that ASD was present in 25.6% of disaster workers, which was significantly higher than that in the unexposed workers (2.4%) [12]. The disaster workers with ASD were 7.3 times more likely to develop subsequent PTSD than those without ASD [12]. A study on the 9/11 World Trade Center attacks reported that 11.1% of disaster workers had probable PTSD, 8.8% had probable depression, 5.0% had probable panic disorder, and 62% had substantial stress reaction [13]. These comorbidities were extensive and associated with high-risk for social impairment [13]. The incidence of posttraumatic psychiatric disorders varies among disaster workers [14]. A study on the Kaprun disaster, a fire that occurred in an ascending train in the tunnel, reported ASD in 25.7% of police officers, 22.2% of crisis intervention workers, and 7.3% of emergency medical personnel [14]. The risk of posttraumatic psychiatric disorders depends on the nature of the disaster, the individual's personality and life history, occupation, and social and psychological support [15].

The most common symptom in this study, that is, recurrent and intrusive distressing recollections of the event, including images, thoughts, or perceptions, that is, reexperiencing, is the core component of ASD and PTSD [9, 16, 17]. Reexperiencing is sudden and unwanted traumatic memories intruding into or even seeming to replace what is happening now, which is usually sensory impression and emotional response from the trauma that appear to lack a time perspective and a context [18]. Intrusive memory can be interpreted as reexperiencing of warning signals [18]. Stimuli that have perceptual similarity to cues accompanying the traumatic event are the trigger of the reexperiencing symptom [18]. The treatment strategies include active incorporation of the updated information into the worst moments of the trauma memory and training of the discrimination between the stimuli during the trauma and trigger of the reexperiencing symptom [18].

There is no study about the comparison of posttraumatic psychiatric disorders following an earthquake disaster between nurses and physicians; however, one study regarding exposure to missile attacks and casualties of war reported that nurses had a higher prevalence of PTSD than physicians, a result similar to the present study [19]. The authors suggested that longer working hours in nurses than in physicians may play a major role [19]; however, it may not be applicable to this study. The present study also showed a nonsignificant higher trend of posttraumatic psychiatric disorders in female HCPs. Previous studies showed that women have higher risk for PTSD than men [20, 21]. Greater fear conditioning in women may be responsible for it [22]. The nonsignificant finding may be due to the small sample size in this study. Further study with more participants is warranted to clarify this issue.

This study showed that a good resilience exists among the HCPs. The possible explanations are early intervention, close subsequent follow-up, and referral for psychiatric counseling as needed in CMMC. Resilience is a key component of maintaining personal health and quality of care in the work place [5]. Several factors affect resilience, including individual (e.g., the capacity for mindfulness and attitudes that promote constructive and healthy engagement with the often-difficult challenges at work), community, and institutional factors [5]. HCPs who care for themselves care for others, including patients, better and make less errors [5]. Resilience-promoting programs are suggested to be implemented among the disaster workers [6]. The National Institute Environmental Health Sciences in the United States suggested the following objectives for training resilience: (1) recognition of the signs and symptoms of disaster work-related stress; (2) obtaining support from the institution and community resources; and (3) building up individual resilience by demonstrating stress reduction and coping strategies [6]. In spite of the positive finding for resilience, the interpretation should be cautious because the definition of resilience in this study has not been validated.

The strength of this study is that it is the first study delineating the incidence of posttraumatic psychiatric disorders and resilience in the HCPs following an earthquake operation with subsequent intervention. Despite this strength, there are some limitations. First, the case number is relatively small, which is limited by the anticipated HCPs. Second, every HCP anticipated only one shift of the medical response (8 h), which may be too short to induce posttraumatic psychiatric disorders as other disaster responses. In other words, the short time of anticipation may not reflect the real impact of the disaster and underestimate the incidence of posttraumatic psychiatric disorders. A prospective cohort study reported that high disaster exposure predicted persistent posttraumatic psychiatric disorders independently [3]. Third, the questionnaire for posttraumatic psychiatric disorders and definition of resilience were made according to the situation of disaster response. Further assessment of the construct and validation for resilience are needed. Fourth, the methodology including the intervention might not be very precise due to the short preparation time for the emergency operation; however, we had done our best to fit the general guideline of Psychological First Aid for Provider Care [4]. Fifth, we could not identify which aspects of intervention helped in reducing the specific symptoms because it is not the scope of this study. Further study is warranted to clarify this issue. Sixth, although the result provides us with an important reference for this issue, it may not be generalized to other circumstances, institutions, occupations, and nations.

## 5. Conclusions

This prospective study delineated that posttraumatic psychiatric disorders were common in the HCPs following the medical response to an earthquake; however, the resilience was good after the early intervention. The most common reported symptom was recurrent and intrusive distressing recollections of the event, including images, thoughts, or perceptions. Nurses and female HCPs had a trend of higher incidence of posttraumatic psychiatric disorders than the physicians. Further studies with more details on psychiatric disorders, other factors affecting resilience, longer duration of the medical response, more severe earthquakes, or different operations are warranted.

## Figures and Tables

**Table 1:** Posttraumatic psychiatric disorders in the HCPs immediately following the medical response to the earthquake.

Variable	Total	Nurses	Physicians
	(*n* = 67)	(*n* = 35)	(*n* = 32)
Age (years)	32.7 ± 5.2	33.5 ± 5.8	32.4 ± 4.4
Age subgroup (years)			
<30	18 (26.9%)	9 (25.7%)	9 (28.1%)
30–39	38 (56.7%)	18 (51.4%)	20 (62.5%)
≥40	11 (16.4%)	8 (22.9%)	3 (9.4%)
Sex			
Male	30 (44.8%)	2 (5.7%)	28 (87.5%)
Female	37 (55.2%)	33 (94.3%)	4 (12.5%)
Years of service	9.0 ± 6.0	11.2 ± 5.9	6.5 ± 5.2
Education			
Bachelor	58 (86.6%)	29 (82.9%)	29 (90.6%)
Master	8 (11.9%)	6 (17.1%)	2 (6.2%)
Ph.D.	1 (1.5%)	0	1 (3.1%)
Marital status			
Married	33 (49.3%)	16 (45.7%)	17 (53.1%)
Unmarried	33 (49.3%)	18 (51.4%)	15 (46.9%)
Divorced	1 (1.5%)	1 (2.9%)	0
Questionnaire (items (1)–(9))^*∗*^			
(1) Recurrent and intrusive distressing recollections of the event, including images, thoughts, or perceptions	9 (13.4%)	7 (20.0%)	2 (6.2%)
(2) Tachycardia	3 (4.5%)	1 (2.9%)	2 (6.2%)
(3) Muscle tension	0	0	0
(4) Difficulty relaxing	3 (4.5%)	2 (5.7%)	1 (3.1%)
(5) Difficulty falling or staying asleep	1 (1.5%)	0	1 (3.1%)
(6) Feeling fear	0	0	0
(7) Feeling guilty	0	0	0
(8) Needing help after the medical response	0	0	0
(9) Needing to talk with someone in private	0	0	0
With posttraumatic psychiatric disorders (any positive item above)	11 (16.4%)	8 (22.9%)	3 (9.4%)

Data are expressed as *n* (%) or mean ± standard deviation. Statistical tests: independent samples *t*-test was used for age and years of service and Pearson chi-square test was used for age subgroup, sex, education, marital status, and questionnaire. ^*∗*^Multiple choices. HCPs, healthcare providers; Ph.D., doctor of philosophy.

**Table 2:** Univariate analysis of posttraumatic psychiatric disorders in the immediate questionnaire in all HCPs.

Variable	With posttraumatic psychiatric disorders (*n* = 11)	Without posttraumatic psychiatric disorders (*n* = 56)	*p* value
Age (years)	33.0 ± 5.7	33.0 ± 5.1	0.975
Age subgroup (years)			>0.999
<30	3 (27.3%)	15 (26.8%)	
30–39	6 (54.5%)	32 (57.1%)	
≥40	2 (18.2%)	9 (16.1%)	
Sex			0.095
Male	2 (18.2%)	28 (50%)	
Female	9 (81.8%)	28 (50%)	
Years of service	10.3 ± 6.3	8.7 ± 6.0	0.436
Occupation			0.191
Nurse	8 (72.7%)	27 (48.2%)	
Physician	3 (27.3%)	29 (51.8%)	
Education			0.719
Bachelor	9 (81.8%)	49 (87.5%)	
Master	2 (18.2%)	6 (10.7%)	
Ph.D.	0	1 (1.8%)	
Marital status			0.551
Married	7 (63.6%)	26 (46.4%)	
Unmarried	4 (36.4%)	29 (51.8%)	
Divorced	0	1 (1.8%)	

Data are expressed as *n* (%) or mean ± standard deviation. Statistical tests: independent samples *t*-test was used for age and years of service and Pearson chi-square test was used for age subgroup, sex, occupation, education, and marital status. HCPs, healthcare providers; Ph.D., doctor of philosophy.

**Table 3:** Basic characteristics of the HCPs with posttraumatic psychiatric disorders following the medical response to the earthquake.

Occupation	Age	Sex	Years of service	Education	Marital status	Number of positive items
Physician	35	M	13	Bachelor	Married	2
Nurse	28	F	7	Bachelor	Married	1
Nurse	41	F	18	Master	Married	1, 4
Nurse	36	F	14	Bachelor	Unmarried	1
Nurse	33	F	11.2	Master	Married	1
Nurse	42	F	20.4	Bachelor	Married	1
Nurse	23	F	1	Bachelor	Unmarried	2
Nurse	33	F	11.3	Bachelor	Married	1
Physician	27	F	2	Bachelor	Married	1
Physician	32	M	4	Bachelor	Unmarried	1, 2, 4, 5
Nurse	33	F	11.1	Bachelor	Unmarried	1, 4

HCPs, healthcare providers; M, male; F, female.
